# 

**Published:** 1847-06

**Authors:** 


ARTICLE XII.

### *New Books.*

We have been notified by Mr. Joseph Keene, successor to Brautigam and Keene, of Chicago, that a number of new works were on the way from the east for us, and also a package from London, care of Wiley and Putnam, New York.

Our readers may expect in the next number of the Journal a full account of all works received.

### NOTICE TO READERS AND CORRESPONDENTS.

We have received original communications from Drs. McNutt, Matthews, and Davis, which will appear in our next.

Those of our correspondents who are not on our regular list, will please to affix their titles to their names—we are often at a loss to do it.

A correspondent wishes to know why our regular contributors do not write more, and why some of them do not write at all.

Received Professor DUNGLISON's Charge to the Graduates of Jefferson Medical College, of Philadelphia, March 25th, 1847 ; with a list of the Graduates, numbering 181.

Catalogue of the Medical Department of Transylvania University for the session of 1846—'47. Number of Students, 205; of Graduates, 68.

Transactions of the Medical Society of the State of New York, vol. vii, part first.

We have received in exchange the following periodicals:

Braithwaite's Retrospect of Practical Medicine and Surgery. Part xiv.
New York Journal of Medicine, etc., New York.
The Annalist, a Record of Practical Medicine, New York.
New York Medical and Surgical Reporter, New York.
The Medical Examiner, etc., Philadelphia.
The Medical News end Library, Philadelphia.
The Western Journal of Medicine and Surgery, Louisville, Ky.
The Western Lancet and Medical Library, Lexington, Ky.
The Southern Medical and Surgical Journal, Augusta, Georgia.
The Boston Medical and Surgical Journal, Boston, Mass.
The Missouri Medical and Surgical Journal, St. Louis, Missouri.
The St. Louis Medical and Surgical Journal, St. Louis, Missouri.
The Practical Educator and Journal of Health, Boston.
Stockton and Co.'s Dental Intelligencer, Philadelphia.

---

### CONTRIBUTORS TO THE ILL. AND IND. MED. AND SURG. JOUR

S. G. Armor, M. D., Rockford, Ill.
A. H. Howland, M. D., Ottawa, Ill.
A. G. Henry, M. D., Pekin, Ill.
Daniel Stahl, M. D., Quincy, Ill.
Edward Mead, M. D., Geneva, Ill.
H. S. Huber, M. D., Chicago, Ill.
David Prince, M. D., Jacksonville, Ill.
P. A. Allaire, M. D., Aurora, Ill.
J. F. Henry, M. D., Burlington, Iowa.
Jno. McLean, M. D., Jackson Mich.
Edward Lewis, M. D., Jackson, Mich.
Ira C. Bachus, M. D., Jackson, Mich.
J. G. Conwell, M.D., Spring Arbor, Mich
S. B. Thayer, M. D., Battle Creek, Mich.
E. Deming, M. D., Lafayette, Ind.
G. N. Fitch, M. D., Logansport, Ind.
G. W. Clippinger, M.D., Terre Haute, Ia
Elias Fisher, M. D. Waynesville, Ohio.

